# Case Report: Rehabilitation assessment and exercise using cardiopulmonary exercise test after coronavirus-disease pneumonia

**DOI:** 10.3389/fresc.2025.1533239

**Published:** 2025-07-04

**Authors:** Ken Kouda, Hideki Konishi, Toshikazu Kubo, Fumihiro Tajima

**Affiliations:** ^1^ Department of Rehabilitation Medicine, Wakayama Medical University, Wakayama, Japan; ^2^ Department of Rehabilitation Medicine, Katsuragi Hospital, Kishiwada, Japan; ^3^ Department of Rehabilitation Medicine, Kyoto Prefectural University of Medicine, Kyoto, Japan; ^4^ Department of Rehabilitation Medicine, Chuzan Hospital, Okinawa, Japan

**Keywords:** cardiopulmonary exercise testing, exercise capacity, COVID-19, physiotherapy, pulmonary rehabilitation, case report

## Abstract

Coronavirus disease (COVID-19) affects not only respiratory function, but also physical function and decreases activities of daily living. Cardiopulmonary evaluation using cardiopulmonary exercise test (CPET) was performed in two patients with persistent respiratory distress one month after acute treatment for COVID-19. The results showed decrease in exercise tolerance, ventilation ability, oxygenation ability, heart rate reserve, stroke volume, and muscle metabolism in both cases. By performing rehabilitation exercise according to the results of CPET, both patients were able to withdraw from oxygen inhalation, improve their functions (17.4 vs. 22.1 in case 1 and 9.0 vs. 16.1 in case 2 on peak exercise aerobic capacity), and were discharged to their homes three months after training. Rehabilitation assessment and exercise using CPET in COVID-19 patients could be safe and useful.

## 1 Introduction

In December 2019, a patient with unexplained pneumonia was reported in Wuhan, China. Since then, the new coronavirus disease (COVID-19) caused by severe acute respiratory syndrome coronavirus 2 (SARS-CoV-2) has spread rapidly worldwide, and reports on rehabilitation medicine for COVID-19 have increased (1, 2). It has been clarified that the elderly and those with underlying diseases such as diabetes and chronic obstructive pulmonary disease are more prone to severe COVID-19. In particular, when elderly people with underlying illnesses suffer from COVID-19, hospitalization treatment is prolonged, and even after discharge, deterioration of respiratory and physical functions hinders daily life. Many patients experienced multiple symptoms, with the majority reporting 5–8 symptoms simultaneously, most associated with fatigue (3). The sequelae of COVID-19 have been investigated, and it was revealed that symptoms such as respiratory distress, malaise, olfaction disorder, cough, and dysgeusia may occur in 5%–18% of cases after two months of its onset, and that the symptoms continue in 2%–11% of cases approximately after four months of onset. It has also been shown that even young people have sequelae (4). In addition, in a cohort study of 1,733 discharged patients, it became clear that 76% of patients had some sequelae even after approximately six months of onset and that more severely ill patients had decreased respiratory function (5). These findings indicate that physical evaluation and rehabilitation exercise are important in preventing the sequelae of COVID-19. Zhao et al. proposed recommendations for rehabilitation of COVID-19 patients from five perspectives: need, practice criteria, remote treatment as needed, importance of evaluation and monitoring, and protection to prevent infection (6). In addition, a study investigating the effects of rehabilitation in the elderly with COVID-19 reported that the group with rehabilitation had significantly improved respiratory function, quality of life, and anxiety after six weeks compared to the group without rehabilitation (7). On the other hand, to analyze, prevent, and improve the mechanism of such sequelae of COVID-19, an exercise load test that can examine exercise tolerance is useful. Exercise load tests included a cardiopulmonary exercise test (CPET), 6-minute walking test, and shuttle walking test. CPET can objectively evaluate maximal oxygen uptake and anaerobic metabolism threshold (AT) by analyzing exhaled gas, and guidelines are published by the American Thoracic Medical Association (ATS) (8). In addition, with CPET, it is possible to prescribe exercise of appropriate intensity to exert a safe and maximum effect from the obtained index, which can be applied to the judgment of the therapeutic effect. In recent years, there have been reports analyzing the causes of impaired exercise tolerance using CPET in patients with sequelae after three months or more than six months after discharge due to COVID-19. However, no reports have demonstrated its usefulness in rehabilitation. In this study, we evaluated cardiopulmonary function using CPET in two patients in six months in Katsuragi Hospital where patients were transferred for rehabilitation after disease or trauma. Those two patients with persistent respiratory distress after one month of acute treatment for COVID-19, and prescribed and implemented exercise according to the index by CPET. As a result, both patients were able to withdraw from oxygen supply, their exercise tolerance improved, and they were discharged from the hospital. We obtained good results and reported the progress of the two cases.

## 2 Case description

**Figure 1:**
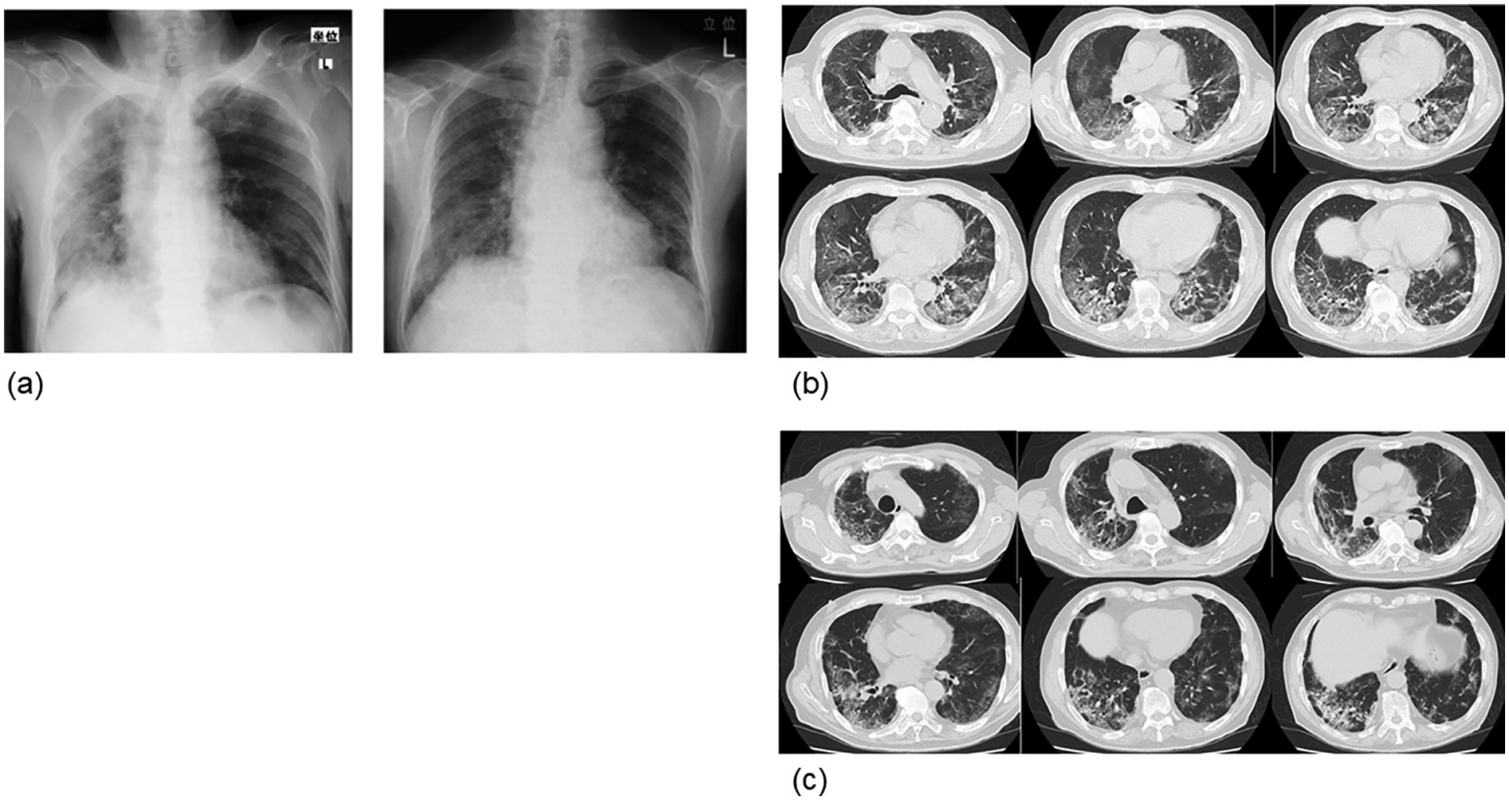
Chest radiograph and computed tomography image (case 1). **(a)** The left is at the time of onset and the right is at the time of admission to our hospital (1 month after onset). Improvement in interstitial inflammatory changes in the right lung was observed, but inflammatory changes persisted in the right lung. **(b)** At the time of onset. Frosted glass-like shadows were observed around the margin, and interstitial inflammatory changes were observed on both dorsal sides. **(c)** One month after admission to our hospital (2 months after onset). The left interstitial inflammation improved, as seen in the chest radiograph, but the interstitial inflammatory changes on both sides, mainly on the dorsal side of the right lung, remained.

Case 1: An 80-year-old man of height 165 cm, weight 64 kg, and body mass index (BMI) 23.7 kg/m^2^ had a complication of essential hypertension, and gastrectomy was performed for gastric cancer 10 years ago. He developed cold symptoms, and approximately a week later, his respiratory condition deteriorated significantly. He visited a medical institution and was diagnosed with COVID-19. Treatment with steroids, favipiravir, and high-flow nasal cannula oxygen alleviated these symptoms. The patient did not require mechanical ventilation. After approximately a month of treatment in an acute care hospital, he was admitted to our hospital for rehabilitation. Activities of daily living (ADL) at admission was almost self-sustaining with functional independence measure (FIM) 124 but required 24-hour oxygen administration (2 L/min); the Borg Scale score increased to 13 and SpO_2_ decreased to 85% after walking for 100 m. Chest radiographs at the acute care hospital and our hospital showed that the interstitial inflammatory changes had improved; however, inflammatory changes persisted in the right lung (Figure 1a). A chest computed tomography (CT) scan at an acute care hospital showed ground glass-like shadows centered on the margin and interstitial inflammatory changes on both dorsal sides (Figure 1b). A chest CT scan at our hospital showed improvement in interstitial inflammatory changes in the left lung, similar to the chest radiographic examination, but inflammatory changes persisted on the dorsal side of the right lung (Figure 1c). Rehabilitation focused on improving thoracic mobility, respiratory muscle training, shoulder girdle relaxation, pectoralis major muscle stretching, sputum drainage, walking training in the prone position, and lower limb ergometer exercise. Rehabilitation evaluation was performed using CPET when walking for 3 minutes. An exhaled gas analyzer (AE-300S, Minato Medical Science Co., Ltd., Osaka, Japan) with a cycle ergometer was used for CPET. A 10-W lamp load test was performed as CPET. The warm-up was performed at 10 W for one minute, increased by 10 W/min, and the end of the load was all-out. In the CPET, oxygen uptake (VO_2_), peak oxygen uptake (peak VO_2_), AT, heart rate (HR), respiratory volume (VE), peak ventilation (peak VE), SpO_2_, and the modified Borg scale were evaluated. In the initial evaluation after one month of its onset, peak VO_2_ was 17.4 ml/kg/min which was lower than the standard value, peak VE was 46.7 L/min, VO_2_/VE at AT was 26.9 ml/L, and peak SpO_2_ was 85%. The peak HR was 137/min and the peak VO_2_/HR was 8.3 (Table 1). The perception of fatigue in the lower limbs was 8 on the modified Borg scale, the exercise duration was 8 minutes 51 s, and the time to reach AT was 7 minutes and 15 s. Based on the above results, as an exercise protocol, exercise was performed using a lower limb ergometer or NUSTEP® (Senoh, Japan), with a frequency of six days per week, intensity of 40%–50% of exercise intensity at AT, and time was set to 20 minutes × 2 times/day. The criteria for discontinuing exercise included a marked decrease in SpO_2_ and exacerbation of subjective symptoms. In the CPET reassessment after two months of starting the training (four months after onset), peak VO_2_ was 22.1 ml/kg/min, peak VE was 56.8 L/min, VO_2_/VE at AT was 26.4 ml/L, and peak SpO_2_ was 89%, all of which increased compared to the initial evaluation. The peak HR was 140/min and peak VO_2_/HR was 10.3, which also increased (Table 1). In addition, the perception of fatigue in the lower limbs was 6 on the corrected Borg scale, and the time to reach AT was 9 minutes and 36 s, both of which improved. The 6-minute walking distance improved to 360 m, 480 m, and 535 m after two (at the time of transfer to our hospital), three, and four months of onset, respectively. No adverse events were reported.

**Table 1:** Results of CPET before and after exercise training in case 1.

Results	Before	After 2 months
VO_2_ max (ml/min)	1,132	1,439
Peak VO_2_ (ml/kg/min)	17.4	22.1
Peak VE (L/min)	46.7	56.8
AT VO_2_/VE (ml/L)	26.9	26.4
Peak SpO_2_ (%)	85	89
Peak HR (beat/min)	137	140
6-minute walking distance (m)	360	535

Before: initial assessment before starting exercise training. After 2 months: evaluation after 2 months of training. CPET, cardiopulmonary exercise test; VO_2_, oxygen consumption; VE, minute ventilation; HR, heart rate; SpO_2_, oxygen saturation.

**Figure 2:**
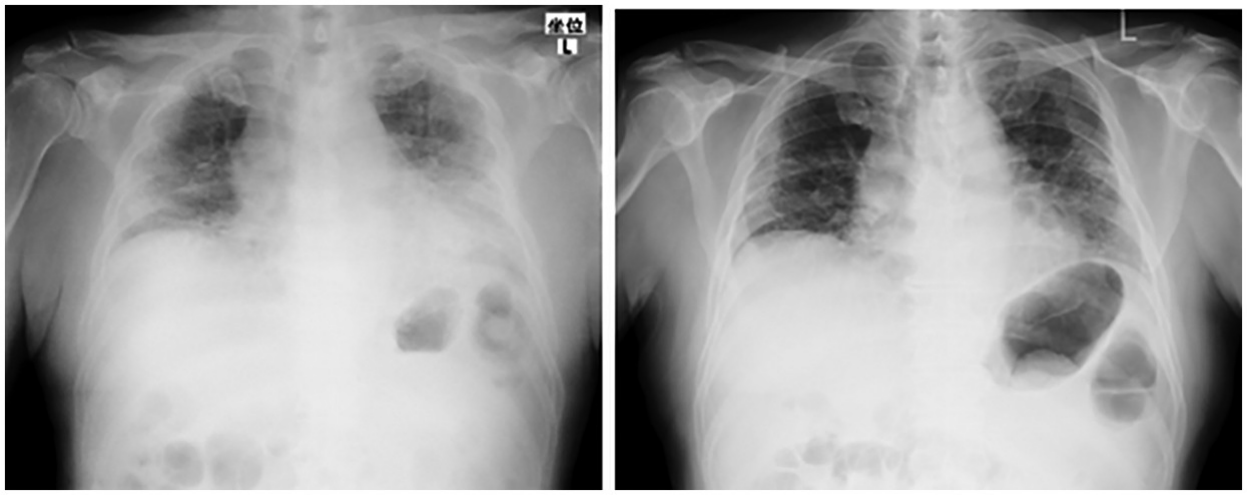
Chest radiograph at the time of onset (case 2). The left side was at the time of admission to our hospital (1 month after onset), and the right was 1 month after admission to our hospital (2 months after onset). Pneumonia was found mainly in the lower lobes on both sides; however, improvement was observed.

Case 2: A 74-year-old man of height 168 cm, weight 62 kg, and BMI 22.1 kg/m^2^ visited a medical institution because of fever and dyspnea and was diagnosed with COVID-19. Steroids and azithromycin were administered, along with high-flow nasal cannula oxygen therapy. The oxygen dose was gradually reduced from 15 L/min to 2 L/min at the time of admission to our hospital, one month after its onset. Disuse due to low activity associated with acute treatment was prominent, and the FIM decreased to 50 after one month of onset. Chest radiographic examination at the time of admission to our hospital showed inflammatory changes mainly in the lower lobes on the left and right, but they improved in approximately one month (Figure 2). Physical evaluation and exercise were performed in the same manner as in case 1, including evaluation using CPET. In the initial evaluation by CPET (Table 2) after one month from onset, the peak VO_2_ was 9.0 ml/kg/min, and the exercise tolerance was extremely low. Peak VE was 28.0 L/min, and VO_2_/VE at AT was 20.3 ml/L. Peak HR was 109/min and peak VO_2_/HR was 5.6, showing a marked decline. The exercise protocol and discontinuation criteria were the same as those in case 1. In the re-evaluation by CPET (Table 2), the peak VO_2_ was 14.3 ml/kg/min, peak VE was 43.3 L/min, and VO_2_/VE at AT was 26.9 after one month from starting the exercise (three months after onset). Two months after starting the exercise (four months after onset), peak VO_2_ was 16.1 ml/kg/min, peak VE was 46.9 L/min, and VO_2_/VE at AT was 28.0 ml/L, and all values increased. One month after starting the exercise, peak HR was 126/min, peak VO_2_/HR was 7.8, and at two months after starting the exercise, peak HR was 120/min and peak VO_2_/HR was 9.4, all of which increased. The 6-minute walking distance at the time of admission to our hospital (one month after the onset) was 80 m while resting under oxygen administration. However, the distance increased over time to 160 m, 230 m, and 345 m after two, three, and four months of onset, respectively. FIM decreased significantly to 50 at the time of admission to our hospital (one month after onset) but improved to 107 after three months and 115 after four months, and the patient was discharged from our hospital. No adverse events were reported.

**Table 2:** Results of CPET before and after exercise training in case 2.

Results	Before	After 1 month	After 2 months
VO_2_ max (ml/min)	606	989	1,127
Peak VO_2_ (ml/kg/min)	9.0	14.3	16.1
Peak VE (L/min)	28.0	43.3	46.9
AT VO_2_/VE (ml/L)	20.3	26.9	28.0
Peak SpO_2_ (%)	76	76	85
Peak HR (beat/min)	109	126	120
6-minute walking distance (m)	80	160	230

Before: initial assessment before starting exercise training. After 1 month and after 2 months: evaluation after 1 month and 2 months of training. CPET, cardiopulmonary exercise test; VO_2_, oxygen consumption; VE, minute ventilation; HR, heart rate; SpO_2_, oxygen saturation.

## 3 Discussion

This case report shows that physical evaluation using CPET and index-based rehabilitation (exercise) could be safe and useful for patients with impaired exercise tolerance due to severe COVID-19. The average alveolar volume in patients with pneumonia decreases in severely ill patients; in COVID-19 patients, it decreases to 87.9%, 80.2%, and 78.9% in mild, moderate, and severe cases (9). In COVID-19, it has been reported that the cytokine storm induces destruction of the alveoli, diffuse alveolar edema, activation of the blood coagulation system, and alveolar septal blood vessel thrombosis, and that not only the decrease in alveolar volume, but also diffuse alveolar damage (DAD) is involved in the abnormal diffusion function (10, 11). With severe COVID-19, respiratory function is significantly impaired, and mechanical ventilation management and high-dose oxygen therapy are required for treatment. In addition, disuse atrophy also occurs due to the effects of long-term bed rest; therefore, even with recovery from COVID-19, ADL decreases. Therefore, rehabilitation for COVID-19 is required from an early stage, but guidelines and protocols have not yet been established (12, 13). In contrast, in severe acute respiratory distress syndrome (ARDS) cases under mechanical ventilation, it has been found that the prone position facilitates successful extubation and shortens the time to extubation (14). In patients with COVID-19, an Italian medical institution investigated whether the prone position could improve oxygenation, and found that repositioning to the prone position improved the PaO2/FiO2 ratio (15). Postural drainage in the prone position is also considered to be effective for lung damage caused by COVID-19. According to the COVID-19 guidelines of the Surviving Sepsis Campaign and the guidelines of the Society of Critical Care Medicine, a prone position for 12–16 hours per day is recommended for patients with COVID-19 undergoing artificial respiration. In addition, in patients with a sedation scale (Richmond Agitation-Sedation Scale) −1 to +1 in the acute phase, lower-limb ergometer exercise including passive exercise is recommended (16). In both of these cases, oxygen therapy was required even one month after the onset, and abnormal findings on chest CT persisted; therefore, sputum drainage in the prone position and lower limb ergometer exercise were performed. This rehabilitation treatment was also considered effective. COVID-19 has sequelae, such as respiratory distress, malaise, cough, and taste/smell disorders, that persist even after discharge. Among these, dyspnea that persisted after six months of discharge was found to be associated with peak VO_2_ using CPET, and decreased physical fitness was found to affect dyspnea (17). In addition, more than a quarter of COVID-19 patients after discharge showed a decrease in ventilation efficiency during exercise, and this decrease was associated with a decrease in heart rate recovery (18). Furthermore, it has been reported that peak VO_2_ was less than 80% of the predicted value in one-third of COVID-19 patients after three months of discharge and that exercise capacity was reduced due to obesity and decreased ventilation efficiency in these patients (19). CPET revealed that COVID-19 has sequelae that affect respiratory function and athletic performance. Exercise-based rehabilitation, including muscle readjustment and respiratory muscle training, is necessary to prevent and improve these sequelae. Hermann et al. reported that they performed cardiac rehabilitation after severe COVID-19 in a patient who was admitted to an acute care hospital, and as a result, the 6-minute walking distance was significantly longer in both the groups with and without the ventilator and reported that rehabilitation was useful in improving physical function (20). Indeed, the COVID-19 patients do not present only with respiratory dysfunctions, but also with cardiological, nutritional, internal and neurological impairments, together with motor difficulties caused by prolonged immobilization. Thus, we must confront the financial and logistical issues since COVID-19 rehabilitation is expensive on cost analysis (21). CPET is also used in rehabilitation for cardiac diseases. Therefore, considered widespread enough in clinical practice. However, frequent measurements during hospitalization are not practical. In addition, patients who have recovered from COVID-19 also exhibit diffusion defect in oxygen delivery and an exaggerated hyperventilatory response during exercise. However, further studies are warranted to investigative the pathobiologic basis of these mechanisms (22). In both cases, when evaluated by CPET at the time of admission to our hospital after one month of onset, peak VO_2_ was low and exercise tolerance was reduced. In addition, SpO_2_ in peak VE and AT was low, and ventilation and oxygenation were also reduced. In addition, peak HR and peak VO_2_/HR were also low, with reduced heart rate reserve and stroke volume. Since the modified Borg scale was large and the duration to reach the AT was less, it was speculated that muscle metabolism was reduced. Evaluation of physical function by CPET is useful, and even if the patient is not a critically ill patient who has been ventilated, if the acute phase is not adequately treated as in these cases, cardiopulmonary and motor functions could decline even at one month after the onset. Therefore, it is considered that even patients with nasal oxygen therapy need rehabilitation exercise for severe ARDS from an early stage. Since the severity and age of COVID-19 patients vary, it is important to plan and implement rehabilitation treatment; however, there is no confirmatory method for each case. Evaluation using CPET is also useful for personalized rehabilitation and has been applied in various fields of cardiac and pulmonary diseases. Exercise therapy was performed based on the CPET results obtained at the time of admission, and in the CPET reassessment after two months from starting the exercise, peak VO_2_ increased and exercise tolerance improved. In addition, peak SpO_2_ increased in peak VE and AT, and ventilation and oxygenation also improved. In addition, peak HR and peak VO_2_/HR also increased, improving the heart rate reserve and stroke volume. The patients were able to withdraw from oxygen, improve their 6-minute gait test, and reach a modified level of independence in their daily life. Improved thoracic mobility, respiratory muscle training, shoulder girdle relaxation, pectoralis major muscle stretching, sputum drainage in the abdominal position, and individual rehabilitation based on CPET evaluation are considered to be safe and useful for functional improvement.

## 4 Conclusions

In this study, cardiopulmonary function evaluation using CPET was performed in two patients with persistent respiratory distress one month after the acute treatment of COVID-19, and it was found that exercise tolerance was reduced. However, exercise therapy according to the results of CPET was able to improve exercise tolerance and the patient was discharged after three months of training. Rehabilitation assessment and exercise using CPET in COVID-19 patients are safe and useful. Future studies should investigate randomized controlled trials to validate CPET-based rehabilitation protocols.

## Data Availability

The raw data supporting the conclusions of this article will be made available by the authors, without undue reservation.
